# Supplementary material for: The Effect of Adding Modified Chitosan on the Strength Properties of Bacterial Cellulose for Clinical Applications
Source: Polymers (Basel). 2021 Jun 18;13(12):1995. doi: 10.3390/polym13121995 (PMC8234744; doi:10.3390/polym13121995)
Supplement: Supplementary file 1 [file polymers-13-01995-s001.zip › s1.pdf]

Novochizol nanoparticles: unique, high yield chemical synthesis

Chitosan

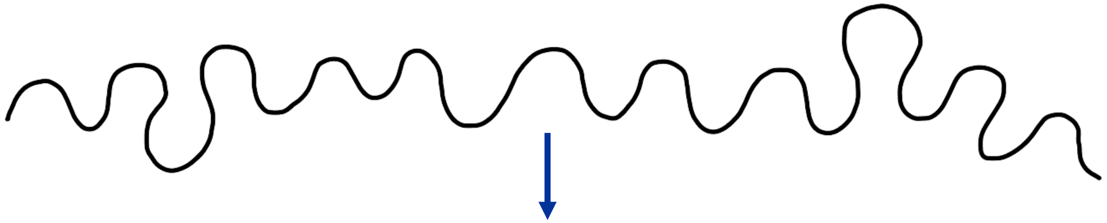

chemical chain cross-linking and folding

Nanoparticle formation

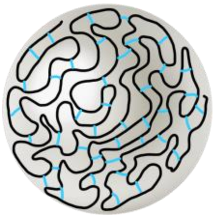

90% yield
